# Comorbidity status in hospitalized elderly in Japan: Analysis from National Database of Health Insurance Claims and Specific Health Checkups

**DOI:** 10.1038/s41598-019-56534-4

**Published:** 2019-12-27

**Authors:** Shuko Nojiri, Hiroaki Itoh, Takatoshi Kasai, Kazutoshi Fujibayashi, Tomoyuki Saito, Yoshimune Hiratsuka, Atsushi Okuzawa, Toshio Naito, Kazuhito Yokoyama, Hiroyuki Daida

**Affiliations:** 1 Medical Technology Innovation Center, Juntendo University, Tokyo, Japan; 2 Department of Epidemiology and Environmental Health, Juntendo University Faculty of Medicine, Tokyo, Japan; 3 Department of Cardiovascular Medicine, Juntendo University Faculty of Medicine, Tokyo, Japan; 4 Department of General Medicine, Juntendo University Faculty of Medicine, Tokyo, Japan; 5 Division of Pharmacy, Juntendo University Hospital, Tokyo, Japan; 6 Department of Ophthalmology, Juntendo University Faculty of Medicine, Tokyo, Japan; 7 Department of Coloproctological Surgery, Juntendo University Faculty of Medicine, Tokyo, Japan

**Keywords:** Diseases, Medical research

## Abstract

The detailed comorbidity status of hospitalized elderly patients throughout Japan has remained largely unknown; therefore, our goal was to rigorously explore this situation and its implications as of the 2015 fiscal year (from April 2015 to March 2016). This study was based on a health insurance claims database, covering all insured policy holders in Japan aged ≥60 years (male: n = 2,135,049, female: 1,969,019) as of the 2015 fiscal year. Comorbidity status was identified by applying principal factor analysis to the database. The factors identified in male patients were [1] myocardial infarction, hypertension, dyslipidemia, and diabetes mellitus; [2] congestive heart failure (CHF), cardiac arrhythmia, and renal failure; [3] Parkinson’s disease, dementia, cerebrovascular disease, and pneumonia; [4] cancer and digestive disorders; and [5] rheumatoid arthritis and hip fracture. However, in female patients, the results obtained for the quaternary and quinary factors were the opposite of those obtained in male patients. In superelderly patients, dementia, cerebrovascular disease, and pneumonia appeared as the tertiary factor, and hip fracture and osteoporosis appeared as the quaternary factor. The comorbidities in the elderly patients suggest the importance of coronary heart disease and its related metabolic disorders; in superelderly patients, fracture and osteoporosis appeared as factors, in addition to dementia and pneumonia.

## Introduction

It is well known that the demographic structure of Japan has been changing dramatically, toward a far more elderly population combined with sharply declining birth rates. The proportion of the population accounted for by the elderly population (aged ≥65 years) will exceed 30% by 2025 and reach a remarkable 40% by 2060. In addition, the elderly population (aged ≥75 years) was 15.60 million (12.3%) in 2013, and it is expected to reach 23.36 million (26.9%) by 2060^1^. Both the World Health Organization and the United Nations define an “aging society” as the one in which >7% of the population is aged ≥65 years, an “aged society” as the one in which >14% of the population is aged ≥65 years, and a “superaged society” as the one in which >21% of the population is aged ≥65 years. The growth rate of the elderly population in Japan is considerably higher than that in other developed countries, partly owing to the advanced medical technology widely available in the country. Japan is now categorized as a superaged society, with >25% of its population aged ≥65 years.

With the growing number of elderly people, the occurrence of age-related diseases has been steadily increasing. Comorbidities are a well-known phenomenon in the elderly population and are associated with a greater risk of death, poor functional status, reduced quality of life, the possibility of a higher risk of adverse events in response to medication and greater use of health-care services^2^. Generally, comorbidities are defined as cooccurring, etiologically independent chronic health conditions, and they are considered an important predictor of survival^3^. It has been reported that quality of life and its relationship with basic and instrumental activities of daily living (ADL) decline with the occurrence of medical illnesses in elderly populations^4,5^. In addition, considerable research has indicated a link of comorbidities with prognostics, quality of life, and health system utilization, such as hospitalization, and a consequent increase in the total costs of health care^6–11^.

In 2008, the National Database of Health Insurance Claims and Specific Health Checkups of Japan (NDB) was created in Japan for the “development, implementation, and evaluation” of the Health Care Cost Containment Plan; this process was followed by the adoption of Section 16 of the Elderly Health Care Security Act^12^. Japan has a universal health-care coverage system comprising National Health Insurance for self-employed and unemployed people, as well as social health insurance for employees and those aged >75 years. A critical feature of this entire system is the use of the Diagnosis Procedure Combination (DPC)/Prospective Payment System (PPS); this term refers to the diagnosis-related group-based prospective payment system, where provider reimbursement is calculated based on a flat-rate per-diem fee, based on the diagnosis group relevant to the service rendered^13^. Currently, data on approximately 7 million inpatients are collected annually, representing approximately 50% of all acute inpatients in Japan in a given year. This database includes the following information: hospital identification code and hospital type, patient data, admission and discharge status, diagnoses, and drugs and procedures used.

Despite the recent emphasis on multiple morbidities, the prevalence of comorbid conditions among the aging patient population, including even the most common combinations of chronic conditions, has remained largely unknown. In particular, knowledge of the prevalence of comorbid conditions in the superelderly society of Japan will provide valuable information for many other nations in which an older distribution of the population will develop soon. According to the patient survey in 2017 in the Ministry of Health, Labour and Welfare in Japan, a large number of Japanese individuals suffered from lifestyle-related diseases, such as hypertension, diabetes, and lipid disorders^14^. Therefore, we hypothesized that cardiovascular diseases, diabetes, and hypertension comprise the core comorbidities in Japanese elderly individuals, as would be shown from an analysis of the national claim database (DPC hospitals) in Japan and that the comorbidity pattern differs by sex in the Japanese superelderly population. To test these hypotheses and to better understand the challenges of coexisting diagnoses in an elderly population, we examined the disease pattern in DPC hospital visits in persons aged ≥60 years in the context of their overall morbidity burden.

## Results

**Figure 1:**
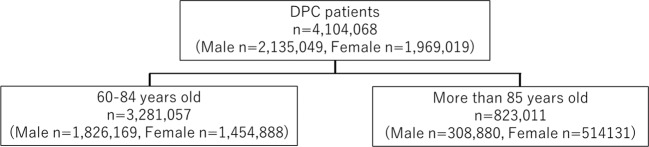
Study population flow. DPC: Diagnosis Procedure Combination. The number of patients for sex (male vs female) and age (60–84 years old vs ≥ 85 years old) stratification are shown.

**Figure 2:**
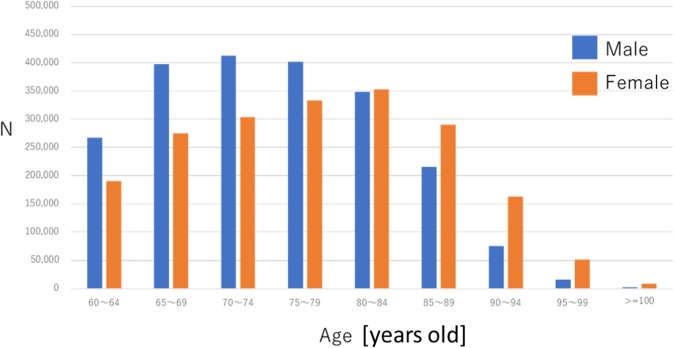
Distribution of the study cohort by age and sex. The age categories are 5-year interval groups in patients aged ≥60 years. Age stratification resulted in nine categories (60–64, 65–69, 70–74, 75–79, 80–84, 85–89, 90–94, 95–99, and ≥100).

**Figure 3:**
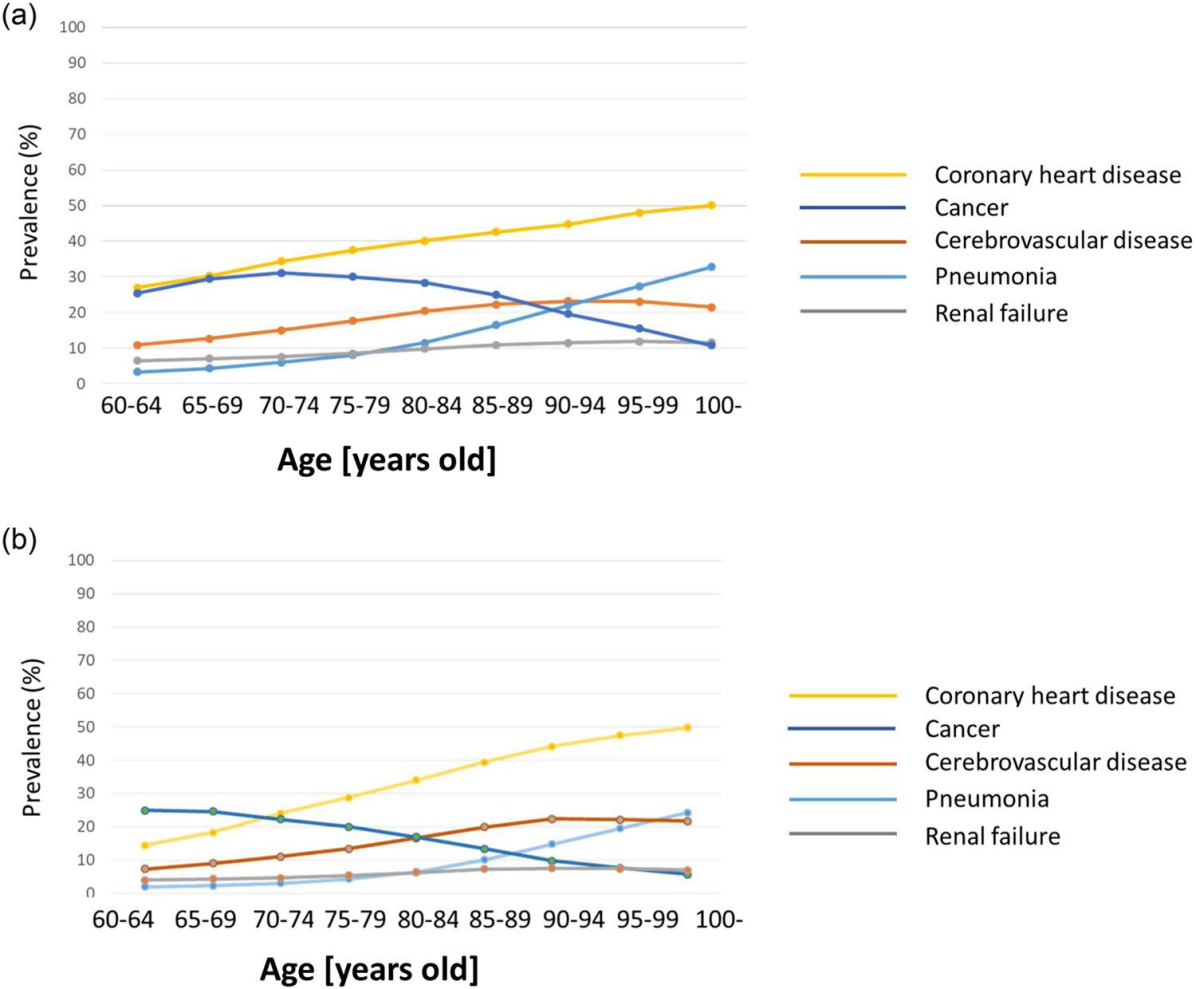
(**a**) Age-stratified distribution of diseases in male patients. The definitions of the diseases are shown in Table 1, indicated with *. Prevalence was calculated by dividing the number of target-disease patients by the total number of patients in the study cohort. **(b)** Age-stratified distribution of diseases in female patients. The definitions of diseases are shown in Table 1, indicated with *. The prevalence was calculated by dividing the number of target-disease patients by the total number of patients in the study cohort.

Our study comprised 2,135,049 male and 1,969,019 female patients aged ≥60 years who had been patients in DPC hospitals during the 2015 fiscal year (Fig. 1). Furthermore, we performed stratified analysis in elderly (60–84 years old) and superelderly (≥85 years old) patients; Fig. 2 shows the distribution of data by age and sex. Different disease statuses showed different prevalence patterns in both male and female DPC hospitalized patients analyzed using disease code in Table 1 (Table 2 and Fig. 3). For pneumonia, a steady upward trend was observed for both sexes. The prevalence of cardiovascular diseases was highest among all ailments in all age groups. Hypertension was the most frequent condition in male and female patients (38% and 37%, respectively; Table 2). Furthermore, diabetes was a remarkably frequent condition (26% in male and 19% in female patients) in addition to cancer.

**Table 1:** Disease definitions.

Disease	ICD10
Cancer^*^	C0XX, C1XX, C2XX, C3XX, C4XX, C5XX, C6XX, C7XX, D0XX
Lymphoma, Leukemia	C08XX, C09XX
Parkinson’s disease	G20, G21X
Dementia	F01X, F03,G30X, G31X
MI	I20XX, I25XX
Acute MI	I21X–X24X
CHF	I50X, I11X
Acute CHF	I509
Cardiac arrhythmia/Atrial fibrillation	I44X–I49X
Artery dissection/Aortic aneurysm	I71X
Hypertension	I10–I15X
Coronary heart disease*	I01X–I51X
Cerebrovascular diseases*	I60X–I69X
Diabetes mellitus	E10X–E14X
Thyroid disease	E00–E07X
Depression	F31X–F34X, F38X
Schizophrenia	F20X,F21X, F231, F232
COPD	J41X–J44X
Pneumonia*	J67X-J70X, J82, J84X–J86X
Dyslipidemia	E78X
Rheumatoid arthritis	M05X, M06X
Knee arthritis	M17X–M19X
Renal failure*	N14X, N17X, Q61X
Hip fracture	S72XX, S73X
Osteoporosis	M80XX, M81XX
Digestive tract disorders	K40X–K46X, K56X
Allergy	H101, H011, H30X, H32X, J45X, J82, K522, L23X, L50X, M35X, M301, T78X, T88X, J450, J82, H101, J301, T887
Cataract	H25X, H26X

^*^Disease presented in Fig. 2.

MI: myocardial  infarction. CHF: congestive heart failure.  COPD: chronic obstructive pulmonary disease

**Table 2:** Prevalence of diseases in the study population.

Elderly individuals (60–84 years old)	Male (n = 1,826,169)		Female (n = 1,454,888)		Sum	
Diseases	n	%	n	%	n	%
Cancer, Solid	522,107	28.6	302,493	20.8	824,600	25.1
Cancer, metastasis	107,603	5.9	64,634	4.4	172,237	5.3
Lymphoma, Leukemia	33,533	1.8	25,251	1.7	58,784	1.8
Parkinson’s disease	25,198	1.4	24,496	1.7	49,694	1.5
Dementia	50,231	2.8	59,328	4.1	109,559	3.3
Myocardial infarction	359,676	19.7	168,825	11.6	528,501	16.1
AMI	340,850	18.7	160,965	11.1	501,815	15.3
CHF	237,953	13	153,058	10.5	391,011	11.9
Acute CHF	17,650	1	10,090	0.7	27,740	0.9
Cardiac arrhythmia	234,961	12.9	139,075	9.6	374,036	11.4
Artery diseases	37,105	2	12,932	0.9	50,037	1.5
Hypertension	694,482	38	504,947	34.7	1,199,429	36.6
Cerebrovascular disease	283,239	15.5	180,706	12.4	463,945	14.1
Diabetes	503,560	27.6	293,336	20.2	796,896	24.3
Diabetes, complicated	164,227	9	92,944	6.4	257,171	7.8
Thyroid diseases	25,109	1.4	40,799	2.8	65,908	2
Hyperlipidemia	343,945	18.8	275,938	19	619,883	18.9
Depression/mood disorder	31,544	1.7	43,540	3	75,084	2.3
Schizophrenia	28,549	1.6	24,634	1.7	53,183	1.6
Pneumonia	122,427	6.7	57,629	4	180,056	5.5
COPD	52,825	2.9	9,176	0.6	62,001	1.9
Digestive tract diseases	362,980	19.9	248,163	17.1	611,143	18.6
Rheumatoid arthritis	16,889	0.9	41,243	2.8	58,132	1.8
Hip fracture	34,286	1.9	53,683	3.7	73,218	2.2
Osteoporosis	19,535	1.1	125,437	8.6	159,723	4.9
Knee arthritis	16,050	0.9	50,055	3.4	66,105	2
Renal failure	145,075	7.9	74,713	5.1	219,788	6.7
Cataract	25,109	1.4	24,383	1.7	49,492	1.5
Allergy	31,924	1.8	26,998	1.9	58,922	1.8
**Super elderly individuals (≥85 years old)**	**Male (n = 308,880)**		**Female (n = 514,131)**		**Sum**	
**Diseases**	**n**	**%**	**n**	**%**	**n**	**%**
Cancer, solid	70,199	22.7	58,423	11.4	128,622	15.6
Cancer, metastasis	10,100	3.3	8,813	1.7	18,913	2.3
Lymphoma, Leukemia	4,113	1.3	4,485	0.9	8,598	1
Parkinson’s disease	6,049	2	9,440	1.8	15,489	1.9
Dementia	37,449	12.1	89,622	17.4	127,071	15.4
MI	54,886	17.8	71,861	14	126,747	15.4
Acute MI	51,538	16.7	67,077	13.1	118,615	14.4
CHF	75,587	24.5	132,243	25.7	207,830	25.3
Acute CHF	3,580	1.2	5,790	1.1	9,370	1.1
Cardiac arrhythmia	53,794	17.4	79,978	15.6	133,772	16.3
Artery diseases	7,325	2.4	6,123	1.2	13,448	1.6
Hypertension	122,878	39.8	228,727	44.5	351,605	42.7
Cerebrovascular diseases	69,660	22.6	109,362	21.3	179,022	21.8
Diabetes	57,433	18.6	78,737	15.3	136,170	16.6
Diabetes, complicated	15,851	5.1	19,559	3.8	35,410	4.3
Thyroid diseases	5,891	1.9	13,488	2.6	19,379	2.4
Hyperlipidemia	36,735	11.9	70,360	13.7	107,095	13
Depression/mood disorder	5,392	1.8	14,320	2.8	19,712	2.4
Schizophrenia	8,451	2.7	14,632	2.9	23,083	2.8
Pneumonia	57,128	18.5	65,944	12.8	123,072	15
COPD	14,252	4.6	3,421	0.7	17,673	2.2
Digestive tract diseases	51,757	16.8	82,162	16	133,919	16.3
Rheumatoid arthritis	2,236	0.7	7,889	1.5	10,125	1.2
Hip fracture	12,057	3.9	62,591	12.2	74,648	9.1
Osteoporosis	8,546	2.8	56,430	11	64,976	7.9
Knee arthritis	3,199	1	12,806	2.5	16,005	1.9
Renal failure	34,177	11.1	38,257	7.4	72,434	8.8
Cataract	1,841	0.6	3,544	0.7	5,385	0.7
Allergy	3,670	1.2	5,066	1	8,736	1.1

AMI: acute myocardial infarction.

CHF: congestive heart failure.

COPD: chronic obstructive pulmonary disease.

The prevalence was calculated by dividing the number of target-disease patients by the total number of patients in the study cohort.

In super elderly patients (≥85 years old), the prevalence of coronary heart disease was the highest (60.3%), followed by that of hypertension and diabetes mellitus (Table 2). The prevalences of cerebrovascular diseases; diabetes mellitus; myocardial infarction; respiratory diseases, such as pneumonia and chronic obstructive pulmonary disease (COPD); and cancer were lower in female patients than in male patients; however, rheumatoid arthritis, hip fracture, and osteoporosis were more prevalent in female patients.

### Principal factor analysis

The scree and variance-explained plots for male patients indicated that four factors were extracted for male and female patients and revealed the Kaiser–Meyer–Olkin (KMO) measure of sampling adequacy value (the plots are not shown). As shown in Table 3, in elderly patients aged 60–84 years, both sexes had some similarities; for example, the prevalence of cancer decreased with age, whereas that of cardiovascular diseases remained stable or increased with age. The primary factor comprised myocardial infarction, hypertension, dyslipidemia, and diabetes mellitus in male patients and hypertension and dyslipidemia in female patients. The secondary factor comprised CHF, cardiac arrhythmia, and renal failure in male patients and CHF and cardiac arrhythmia in female patients. The tertiary factor comprised Parkinson’s disease, dementia, cerebrovascular disease, pneumonia, and dementia in female patients. The quaternary factor comprised digestive disorders and cancer in male patients and hip fracture in female patients. The quinary factor comprised rheumatoid arthritis and hip fracture in male patients and cancer and digestive tract diseases in female patients.

**Table 3:** Factor loading.

	Factor 1	Factor 2	Factor 3	Factor 4	Factor 5
**Male**					
Cancer	−0.10659	−0.05222	−0.09336	**0.29196**	−0.00884
Parkinson’s disease	−0.02062	−0.03614	**0.21814**	−0.01222	−0.00269
Dementia	−0.02648	0.01538	**0.32115**	0.00136	0.00155
Myocardial infarction	**0.42249**	0.13297	−0.12004	−0.04151	0.01509
CHF	0.05612	**0.47085**	−0.01372	0.0048	0.00475
Cardiac arrhythmia	−0.02745	**0.37216**	0.01214	0.01618	−0.08424
Artery diseases	0.07138	0.07921	−0.00823	0.01965	−0.02685
Hypertension	**0.47679**	0.06879	0.08519	0.09994	−0.00291
Cerebrovascular disease	0.15291	−0.0721	**0.29319**	−0.02358	−0.09107
Diabetes mellitus	**0.29548**	−0.0448	−0.00527	−0.00833	0.09277
Thyroid diseases	0.01417	0.11175	0.0111	0.04953	0.06683
Dyslipidemia	**0.57063**	−0.06311	−0.03038	0.00673	−0.01019
Depression/mood disorder	0.02248	0.00256	0.12016	0.09888	0.02821
Schizophrenia	−0.01349	0.02271	0.18805	0.10024	0.01356
Pneumonia	−0.07223	0.06967	**0.3131**	0.03064	0.06878
COPD	−0.06154	0.11722	0.02537	0.06022	0.05191
Digestive tract disorders	0.06764	0.00467	0.02184	**0.27813**	0.01561
Rheumatoid arthritis	0.03275	0.01494	0.00835	−0.02695	**0.21007**
Hip fracture	0.05807	0.02472	0.0555	−0.00443	**0.25913**
Osteoporosis	−0.01067	−0.00646	0.10256	−0.06517	0.08753
Knee arthritis	0.01014	−0.02395	−0.01496	−0.06566	0.05874
Renal failure	0.03218	**0.20559**	−0.00659	−0.02431	0.09561
Cataract	−0.02855	−0.0711	−0.0536	−0.13754	0.02248
Allergy	0.01982	0.00597	−0.00043	0.02447	0.06574
**Female**					
**Diseases**	**Factor 1**	**Factor 2**	**Factor 3**	**Factor 4**	**Factor 5**
Cancer, Solid	−0.04696	−0.07181	−0.08844	−0.09875	**0.22479**
Parkinson’s disease	−0.01194	−0.02114	0.13268	0.00562	0.01039
Dementia	−0.00599	0.01341	**0.25166**	0.00006	−0.02855
Myocardial infarction	0.16585	0.12614	−0.07092	0.01259	−0.00285
CHF	0.00204	**0.34171**	0.02537	−0.02638	−0.00008
Cardiac arrhythmia	0.00029	**0.24956**	0.00278	−0.05825	−0.02486
Artery diseases	0.01877	0.05125	−0.00518	−0.00953	0.00431
Hypertension	**0.29353**	0.07467	0.0658	0.04247	0.06172
Cerebrovascular diseases	0.10895	−0.00564	0.16191	−0.10327	−0.07211
Diabetes	0.14761	0.0035	−0.03406	0.00243	0.01243
Thyroid diseases	0.01013	0.05013	0.00308	0.033	0.0807
Dyslipidemia	**0.30281**	−0.01597	−0.04947	0.03593	0.02577
Depression/mood disorder	0.00892	−0.0188	0.09625	0.03691	0.08107
Schizophrenia	−0.01929	−0.00328	0.14341	0.01503	0.06187
Pneumonia	−0.0425	0.04353	0.19937	−0.03839	0.00604
COPD	−0.02339	0.03435	0.00834	0.01295	0.04067
Digestive tract diseases	0.02156	−0.01016	0.01498	−0.01643	**0.20743**
Rheumatoid arthritis	−0.02619	0.01726	−0.00203	0.17131	0.03369
Hip fracture	0.02699	−0.01392	0.05146	**0.24698**	0.03818
Osteoporosis	−0.00923	−0.01101	0.1114	0.14019	−0.07293
Knee arthritis	0.02334	−0.02473	−0.03585	0.11474	−0.05217
Renal failure	−0.00185	0.13512	−0.01153	0.00353	0.01609
Cataract	−0.00185	−0.02865	−0.03863	0.0049	−0.0796
Allergy	0.00401	−0.00542	−0.00916	0.0397	0.04529

Factor loadings > 0.20 are highlighted in bold.

CHF: congestive heart failure.

COPD: chronic obstructive pulmonary disease.

Male: KMO = 0.641.

Female: KMO = 0.617.

### Factor analyses in age categories

In elderly patients (60–84 years old), the scree and variance-explained plots for male patients indicated that the numbers of factors extracted were four for male patients and three for female patients (the plots are not shown). As shown in Table, the primary factor for male patients comprised myocardial infarction, dyslipidemia, diabetes mellitus, and hypertension, whereas for female patients, the primary factor comprised the above diseases in addition to cerebrovascular diseases. The secondary factor comprised CHF, followed by cardiac arrhythmia for both sexes. The tertiary factor comprised cerebrovascular diseases for male patients, whereas it comprised Parkinson’s disease, dementia, schizophrenia, and pneumonia for female patients. The quaternary factor comprised rheumatoid arthritis and hip fracture, and the quinary factor comprised cancer and digestive tract diseases for female patients. In contrast, the quaternary factor comprised cancer and digestive tract diseases, and the quinary factor comprised rheumatoid arthritis and hip fracture for male patients.

**Table 4:** Factor loading.

Diseases	Factor 1	Factor 2	Factor 3	Factor 4	Factor 5
**Male elderly patients (60–84 years old)**					
Cancer	−0.1024	−0.06721	−0.08211	−0.02403	**0.29578**
Parkinson’s disease	−0.02787	−0.01375	**0.21532**	0.01768	0.0003
Dementia	−0.01081	0.01896	**0.2981**	0.01518	0.02855
Myocardial infarction	**0.51121**	0.04535	−0.14083	0.03175	−0.04289
CHF	0.10847	**0.42914**	−0.0156	0.01455	0.01448
Cardiac arrhythmia	−0.03568	**0.35683**	−0.01078	−0.09536	−0.02306
Artery diseases	0.08745	0.04885	−0.00743	−0.029	0.02293
Hypertension	**0.47728**	0.08384	0.09433	−0.0317	0.1055
Cerebrovascular diseases	0.11878	−0.08851	**0.24779**	−0.13518	−0.05801
Diabetes mellitus	**0.28235**	−0.01163	0.02272	0.0998	0.01075
Thyroid diseases	0.01805	0.10971	0.01921	0.05661	0.05164
Dyslipidemia	**0.61012**	−0.07227	−0.02452	−0.01374	0.00987
Depression/mood disorder	0.02655	0.01031	0.14006	0.01598	0.1198
Schizophrenia	0.00177	0.0257	0.19847	0.00741	0.13003
Pneumonia	−0.04009	0.03621	0.2797	0.11846	0.01708
COPD	−0.03245	0.08027	0.02807	0.06439	0.04053
Digestive tract diseases	0.04021	−0.00857	0.02727	−0.01545	**0.23908**
Rheumatoid arthritis	0.03268	−0.01079	0.01752	**0.21883**	−0.06772
Hip fracture	−0.01309	−0.00524	0.08795	0.07843	−0.04836
Osteoporosis	0.05722	0.00557	0.07358	**0.27044**	−0.0339
Knee arthritis	−0.01291	−0.02644	−0.02123	0.05175	−0.0882
Renal failure	0.0647	0.18047	0.02112	0.1019	0.00667
Cataract	−0.06291	−0.0478	−0.05122	0.03495	−0.15322
Allergy	0.00938	0.00366	0.0121	0.07502	−0.00008
**Female elderly patients (60–84 years old)**					
Cancer	−0.06801	−0.07115	−0.05775	−0.08756	**0.28466**
Parkinson’s disease	−0.02227	−0.02213	**0.23825**	−0.00431	−0.00178
Dementia	0.01882	0.00341	**0.32325**	−0.02986	−0.01322
Myocardial infarction	**0.28603**	0.19323	−0.10112	−0.01405	−0.01966
CHF	0.01464	**0.49874**	0.00647	−0.00099	0.02306
Cardiac arrhythmia	−0.0172	**0.40513**	−0.00094	−0.03941	−0.00846
Artery diseases	0.02798	0.1124	−0.00542	−0.00981	0.00705
Hypertension	**0.48647**	0.0729	0.05205	0.02556	0.07048
Cerebrovascular diseases	**0.21894**	−0.03493	0.19468	−0.10774	−0.07056
Diabetes	**0.27891**	0.00241	−0.01274	−0.0069	0.00997
Thyroid diseases	0.02791	0.08459	0.0171	0.06042	0.09894
Dyslipidemia	**0.51086**	−0.03947	−0.05489	0.01632	0.01224
Depression/mood disorder	0.02689	−0.02188	0.17483	0.02335	0.08925
Schizophrenia	−0.0178	−0.0006	**0.23493**	−0.01643	0.07704
Pneumonia	−0.0637	0.07172	**0.24735**	0.038	0.04559
COPD	−0.04373	0.07151	0.02041	0.03768	0.05997
Digestive tract diseases	0.0627	0.00663	0.04603	0.02589	**0.26809**
Rheumatoid arthritis	−0.06696	0.03746	−0.00429	**0.27322**	0.0343
Hip fracture	0.03756	−0.02941	0.05048	**0.31976**	0.04199
Osteoporosis	−0.00866	−0.03176	0.14316	0.09694	−0.07701
Knee arthritis	0.03479	−0.06645	−0.06642	0.14005	−0.08915
Renal failure	0.01721	**0.20968**	0.0115	0.00902	0.01943
Cataract	−0.02398	−0.05107	−0.05335	−0.01588	−0.11745
Allergy	0.00229	−0.00161	−0.01016	0.06523	0.0506
**Male super elderly patients (**≥**85 years old)**					
Cancer	−0.00454	−0.12198	−0.26814	−0.01726	−0.08627
Parkinson’s disease	−0.01105	−0.03181	0.04739	0.13448	−0.0103
Dementia	−0.02021	−0.00042	0.08923	**0.2642**	0.0119
Myocardial infarction	**0.40821**	0.0717	−0.0718	−0.00175	0.00662
CHF	0.04969	**0.44438**	0.00075	0.02155	−0.03079
Cardiac arrhythmia	−0.02461	**0.30865**	0.10357	−0.05767	−0.06855
Artery diseases	0.10882	−0.00453	−0.03224	0.00834	−0.0053
Hypertension	**0.36514**	0.08195	0.07908	0.03971	−0.00074
Cerebrovascular disease	0.06673	−0.14814	**0.31551**	−0.02048	−0.05934
Diabetes	0.20685	−0.02931	0.022	−0.01754	0.03759
Thyroid diseases	0.00491	0.13504	−0.02796	0.05055	0.01699
Dyslipidemia	**0.48848**	−0.0656	0.02497	0.00202	−0.00268
Depression/mood disorder	0.05996	0.01683	−0.08021	0.17694	−0.00062
Schizophrenia	0.0271	0.03889	−0.06776	**0.23789**	−0.01022
Pneumonia	−0.11144	0.00524	0.10856	**0.22404**	−0.02484
COPD	−0.03803	0.08954	−0.07628	0.07084	−0.01389
Digestive tract diseases	0.07647	−0.0585	−0.14679	0.07073	−0.07551
Rheumatoid arthritis	0.01821	0.00084	0.0239	−0.01945	0.13312
Hip fracture	−0.00103	−0.00299	0.0356	0.02722	0.19791
Osteoporosis	0.04611	0.00493	0.0152	0.00573	**0.21307**
Knee arthritis	0.00706	−0.0089	0.0385	−0.06937	0.09256
Renal failure	0.0026	**0.2062**	−0.03238	−0.01209	0.04721
Cataract	−0.01018	−0.02723	0.0292	−0.05883	0.03832
Allergy	0.05027	0.0176	−0.03735	0.05576	0.02405
**Female super elderly patients (**≥**85 years old)**					
Cancer	−0.02859	−0.01918	−0.08601	−0.04066	**0.2555**
Parkinson’s disease	−0.03518	0.00656	0.15987	0.04229	0.03776
Dementia	−0.03019	−0.00852	**0.28501**	0.12181	−0.04455
Myocardial infarction	**0.25026**	0.1504	−0.07021	0.05577	0.03292
CHF	0.08525	**0.40035**	0.01792	−0.0348	−0.05098
Cardiac arrhythmia	0.10135	**0.24328**	−0.01199	−0.1123	−0.09931
Artery diseases	0.04632	0.03205	−0.01086	−0.00602	0.012
Hypertension	**0.4401**	0.03194	0.04861	0.08857	0.02521
Cerebrovascular diseases	0.14035	−0.12812	0.17676	−0.17627	−0.06381
Diabetes	0.17955	−0.00041	−0.05278	−0.01054	0.01291
Thyroid diseases	0.04727	0.12521	0.04429	0.05784	0.07531
Dyslipidemia	**0.41815**	−0.06146	−0.05579	0.03658	0.03449
Depression/mood disorder	0.02356	0.05137	0.13177	0.15617	0.06861
Schizophrenia	−0.04456	0.08244	0.1824	0.17289	0.0298
Pneumonia	−0.09183	0.03422	**0.29475**	−0.07629	0.02577
COPD	−0.01875	0.05721	0.02131	0.01096	0.02394
Digestive tract diseases	0.09171	0.03716	0.06605	0.02802	**0.24669**
Rheumatoid arthritis	0.02513	0.00928	−0.00068	0.06674	0.01955
Hip fracture	0.14279	−0.06959	0.00195	**0.22487**	−0.0212
Osteoporosis	−0.03722	−0.06639	−0.01638	**0.26974**	−0.19045
Knee arthritis	0.06269	−0.04772	−0.05782	0.03702	−0.01893
Renal failure	−0.01121	**0.24236**	−0.03035	0.01808	0.01661
Cataract	0.01009	−0.04085	−0.0413	0.00368	−0.02074
Allergy	0.04711	0.01675	0.02764	0.05616	0.0587

Factor loadings >0.20 are highlighted in bold.

CHF: congestive heart failure.

COPD: chronic obstructive pulmonary disease.

Male elderly patients (60–84 years old): KMO = 0.649.

Female elderly patients (60–84 years old): KMO = 0.6333.

Male super elderly patients (≥85 years old): KMO = 0.605.

Female super elderly patients (≥85 years old): KMO = 0.576.

In super elderly patients, the scree and variance-explained plots indicated that the number of factors extracted was three for male patients and five for female patients. For male patients, the primary factor comprised myocardial infarction, dyslipidemia, diabetes and hypertension. For females, the primary factor comprised myocardial infarction, dyslipidemia, and hypertension, whereas the secondary factor comprised CHF, followed by cardiac arrhythmia and renal failure; the secondary factor was the same in both sexes. The tertiary factor for male patients comprised pneumonia, followed by dementia and cerebrovascular diseases, whereas for female patients, it comprised pneumonia and dementia. The quaternary factor comprised only osteoporosis in male patients and comprised hip fracture and osteoporosis in female patients. The quinary factor comprised cancer and digestive tract diseases in female patients.

### Age- and sex-adjusted odds ratios

Table 5 shows the age- and sex-adjusted odds ratios for comorbidities. High odds ratios were observed for the following diseases: acute myocardial infarction and CHF, acute myocardial infarction and dyslipidemia, cardiac arrhythmia and CHF, myocardial infarction and artery dissection, acute CHF and artery dissection, dementia and Parkinson’s disease, dementia and schizophrenia, hypertension and dyslipidemia, pneumonia and rheumatoid arthritis, pneumonia and dementia, hip fracture and schizophrenia, osteoporosis and rheumatoid arthritis, depression and Parkinson’s disease, depression and schizophrenia, Parkinson’s disease and schizophrenia, and cancer and digestive tract diseases.

**Table 5:** Age- and sex-adjusted odds ratios for comorbidities.

Event→	Cata	AMI	MI	CHF	CVD	Dem	COPD	HT	Pneu	a CHF	Alle	RF	HF	CA	KA	RA	Dep	Schizo	Osteo	PD	DL	AD	DD	TD	Cancer	DM
Cata																										
AMI	0.47																									
MI	0.44	**>999**																								
CHF	0.45	**5.53**	**3.67**																							
CVD	0.56	0.70	0.84	1.02																						
Dem	0.54	0.79	0.73	0.96	**1.77**																					
COPD	0.46	0.69	0.87	**1.71**	0.62	0.87																				
HT	0.59	**3.36**	**2.99**	**2.44**	**2.16**	1.17	**1.02**																			
Pneu	0.44	0.68	0.60	**1.20**	0.66	**2.58**	**2.33**	0.82																		
a CHF	0.41	**9.43**	**4.16**	**>999**	1.00	0.87	**1.35**	**2.23**	1.14																	
Alle	**1.26**	0.69	0.87	1.01	0.77	0.86	**2.10**	**1.21**	**1.46**	0.82																
RF	0.54	**1.55**	**1.76**	**2.96**	1.02	1.05	0.73	**1.80**	0.98	**2.64**	1.00															
HF	0.70	0.50	0.82	0.66	0.77	**1.92**	0.72	1.12	0.57	0.64	0.84	0.87														
CA	0.44	**2.21**	**1.88**	**5.10**	**1.55**	0.88	1.02	**2.02**	0.97	**3.17**	0.86	**1.43**	0.69													
KA	0.70	0.86	0.78	0.69	0.63	0.62	0.52	**1.27**	0.41	0.64	**1.35**	0.66	0.79	0.74												
RA	0.63	0.86	0.82	1.04	0.70	0.75	**1.64**	1.03	**2.49**	1.04	**1.35**	1.06	**1.31**	0.76	**1.64**											
Dep	0.62	0.74	0.87	1.02	**1.16**	**2.16**	**1.40**	**1.23**	**1.64**	1.03	**1.47**	0.95	**1.83**	0.88	1.06	1.07										
Schizo	0.46	0.93	0.71	1.13	**1.21**	**3.78**	**1.28**	1.00	**2.33**	1.13	1.11	0.99	**2.62**	0.90	0.78	0.86	**4.77**									
Osteo	0.86	0.60	0.89	0.88	0.81	1.12	**1.41**	**1.62**	**1.38**	0.73	**1.92**	1.04	**2.39**	0.76	**2.23**	**5.21**	**1.57**	1.15								
PD	0.44	0.51	0.55	0.77	**1.49**	**3.63**	0.51	0.82	**3.72**	0.66	0.81	0.67	**1.61**	0.66	0.68	0.64	**3.51**	**3.95**	**1.38**							
DL	0.62	**7.03**	**1.66**	**1.99**	**1.68**	0.83	0.79	**5.18**	0.58	**2.11**	1.16	1.07	0.79	**1.56**	1.15	0.91	1.06	0.69	**1.43**	0.63						
AD	0.35	1.13	**2.48**	**2.53**	**1.12**	0.83	**1.54**	**3.23**	0.74	**7.69**	0.75	**1.80**	0.54	**1.49**	0.40	1.05	0.92	0.99	0.68	0.47	**2.08**					
DD	0.47	1.10	0.91	0.96	0.99	0.93	0.99	**1.29**	1.08	**1.20**	**1.29**	0.97	0.61	0.95	0.77	**1.22**	**1.36**	**1.40**	**1.30**	0.91	1.03	1.13				
TD	0.63	1.07	**1.29**	0.95	0.95	1.14	1.09	**1.71**	**1.24**	**1.41**	**1.48**	**2.31**	0.82	**1.73**	0.89	**1.43**	**1.58**	**1.24**	**1.65**	1.27	**1.57**	1.10	**1.31**			
Cancer	0.31	0.28	0.44	0.46	0.42	0.57	**1.23**	0.73	0.75	0.57	0.94	0.60	0.34	0.57	0.30	0.54	1.15	**1.21**	0.67	0.40	0.52	0.45	**1.88**	1.05		
DM	**1.30**	**1.67**	**2.00**	**1.48**	**1.30**	1.03	0.78	**1.97**	1.05	**1.52**	0.98	**1.90**	1.09	1.10	1.07	1.11	0.98	0.84	1.06	0.71	**2.08**	0.73	1.88	**1.45**	0.85	

Cata = Cataract, AMI = Acute myocardial infarction, MI = Myocardial infarction, CHF = Congestive heart failure, CVD = Cerebrovascular diseases, Dem = Dementia, COPD = Chronic obstructive pulmonary disease, HT = Hypertension, Pneu = Pneumonia, a CHF = Acute congestive heart failure, Alle = Allergy, RF = Renal failure, HF = Hip fracture, CA = Cardiac arrhythmia, KA = Knee arthritis, RA = Renal failure, Dep = Depression, Schizo = Schizophrenia, Osteo = Osteoporosis, PD = Parkinson’s disease, DL = Dyslipidemia, AD = Artery dissection, DD = Digestive disorders, TD = Thyroid disease, DM = Diabetes mellitus.

Bold numbers indicate a risk of >1.2.

## Discussion

This study is the first to examine comorbidities in elderly (aged ≥60 years) patients using routine claims data from the Japanese National Database for health-care information. The major findings of the present study are as follows: (1) the highest prevalence rates (>20%) in elderly patients in Japan were found for cancer, coronary diseases, and diabetes mellitus in DPC hospitals; (2) a downward trend in the prevalence rate of cancer was observed, but an upward pattern was evident for pneumonia with age, with cardiovascular diseases showing a high prevalence across all age groups; (3) in our exploratory factor analysis, cardiovascular diseases combined with diabetes mellitus constituted a primary factor, followed by cerebrovascular diseases, depression, and pneumonia as the secondary factor and cancer and digestive disorders or osteoporosis and rheumatoid arthritis as the tertiary factor; (4) a strong association was observed within some disease combinations such as myocardial infarction, dyslipidemia, and hypertension; Parkinson’s disease, pneumonia, and dementia; hip fracture and schizophrenia; and hip fracture and osteoporosis; and (5) in terms of the superelderly patient disease pattern, the comorbid status for the primary and secondary factors was the same as that in the elderly patients (60–84 years old); however, osteoporosis and hip fracture or rheumatoid arthritis appeared in super elderly patients.

Regarding characteristics of comorbidity status in elderly patients in Japan, by quantifying the prevalence of comorbid conditions at specific ages, our results offer a starting point toward characterizing elderly patients with medically complex conditions. We confirmed that metabolic diseases such as hypertension, diabetes, and cardiovascular diseases, in addition to neurological diseases, including dementia, cerebrovascular disease, pneumonia, hip fracture, and cancer, account for the major health burden for elderly patients in Japan^15^. The results of this study largely concur with the findings of earlier research in other parts of the world^16^, which has identified clear relationships for three comorbidity patterns: cardiovascular and metabolic diseases, mental health problems, and musculoskeletal diseases. The high prevalence of various chronic diseases, including diabetes, has shown an age-dependent increase in younger Japanese patients as well, underlining the importance of disease prevention to avoid unnecessary complications^17^. In addition, we observed that 38% of all patients had hypertension and confirmed the widely held view that hypertension is closely related to cardiovascular diseases and diabetes. Several studies in Western nations found a similar cluster of cardiometabolic diseases to be the primary cause of health challenges in the elderly^18–22^. Cardiovascular and metabolic disorders, a grim partnership that has long been known to be a major driver of low health status and high social costs, are primarily characterized by insulin resistance, hypertension, and obesity. Our study results were similar to Bahat’s *et al*.^23^ results, which showed that cardiovascular diseases were the primary diseases in the elderly. One implication from this study may include that physicians should oversee the early prevention of cardiovascular diseases, in addition to neurological disorders, pneumonia, hip fracture, and cancer.

Our study results reflect the experience of elderly patients at a relatively severe or terminal stage of health problems and complications because it included only DPC inpatients who needed secondary care for special procedures, treatments, or surgeries. For example, the prevalence of pneumonia increases with age, whereas that of cancer decreases with age after a point; in contrast, the prevalence of cardiovascular diseases becomes relatively stable after a substantial increase in the 60- and 70-year age brackets. We did not evaluate the age-adjusted incidence rate of cancer; however, the burden of cancer remained high in the superelderly patients (solid cancer: male, 22.7% and female, 11.4%) (Table 2). These data indicate that Japan will face a substantial increase in the number of elderly patients with cancer, which is consistent with the findings of other reports^24,25^. A more detailed analysis, such as year trend analysis including cancer sites and geographical distribution, will provide further information.

Regarding Parkinson’s disease and its related diseases, several studies have demonstrated that age influences their clinical progression. Aging was found to be directly related to faster motor progression, decreased levodopa responsiveness, severe gait and postural impairment, severe cognitive impairment, and the development of dementia^26–30^ in patients with Parkinson’s disease. Our results showed a strong association of Parkinson’s disease with dementia (age-adjusted odds ratio = 3.63) in addition to mental disorders, such as depression and schizophrenia. In addition, neurological diseases and mental disorders were found to be secondary factors in the elderly female patients included in this study. In male patients, cerebrovascular diseases were in the same category as cardiometabolic diseases. While many earlier investigations from different countries have shown that cardiac insufficiency is a risk factor for stroke, which in turn increases the risk for vascular dementia^31,32^, our data revealed a correlation with dementia (age- and sex-adjusted odds ratio = 1.77), and this correlation was found only in the factor analysis of elderly male patients (Table 4). Our study also found a prominent combination of pneumonia and mental illness, which is explainable by reports showing that patients with dementia and other neurological diseases present with functional and cognitive impairment for a long period before death and may also develop dysphasia, nutritional deficiency, pneumonia, and immobility, therefore becoming partially or completely dependent for their ADL^33–36^.

We found considerable differences in the patterns of comorbidities between sexes. The prevalence rate of cardiovascular diseases (e.g., acute myocardial infarction, diabetes mellitus, cerebrovascular diseases, aortic dissection) was found to be higher in male patients than in female patients (60–64 years old). However, this sex difference disappeared in superelderly patients. Furthermore, the prevalence rate of musculoskeletal diseases, such as rheumatoid arthritis, hip fracture, and osteoporosis, was higher in female patients. This finding can be explained by the fact that women survive longer and exhibit higher rates of mechanical disorders, as suggested by Abad-Diez, J. M.^37^. The female pattern of hip fracture, osteoporosis, and rheumatoid arthritis, which did not appear in elderly male patients, suggested an association between mental disorders and frailty, especially regarding hip fractures in female patients. Sex differences in prevalence rates might account for the different compositions of these patterns, e.g., the combination of rheumatoid arthritis and hip fracture was mostly found in females^38,39^. This disease pattern can be explained by sex hormones; estrogens, along with prolactin, are associated with a female predisposition to autoimmune and rheumatic diseases, and sex hormones are related to the upregulation of the expression of adiponectin levels, which reduces the risk of type 2 diabetes in females^40,41^. Women were affected more frequently by dementia, depression, osteoarthritis, rheumatic diseases, and hip fracture, which are responsible for further impairment of daily life activities, and the identification of these differences could represent a powerful tool to promote sex-specific personalized care in elderly inpatients.

This article’s results should also be interpreted while keeping in mind some other limitations. First, our dataset did not include the outpatient claim diagnosis, which could not be validated via clinical examinations. In our study, the results can be readily explained by how we identified comorbidity patterns considering the characteristics of the large DPC database, where inpatients constituted a relatively large proportion of all the acute phase patients; therefore, caution is required when interpreting the results because they do not precisely reflect what would be seen in a broader primary care population. Generally, the claim data for diagnosis comprise the diagnosis for reimbursement; in addition, physician-related variations may exist in reporting comorbid conditions in claim data. Therefore, in our study, we were able to take our analysis only to the limits of the DPC data because the DPC records are more accurate due to medical institutions usually being subject to a review of their requests for insurance payments; furthermore, Yamana *et al*.^38^ reported the validity of the claim data in DPC hospitals. In addition, our data did not include information on outpatient prescriptions, and therefore, the analyses likely underestimate the extent of the morbidity burden as well as the degree of the comorbidities. Because we used claim data for the main or secondary disease diagnosis, we assume that our data do not reflect the full range of comorbid conditions. Second, elderly individuals with no diseases or any outpatient status were not included in our study, which might lead to the overestimation of correlations among diagnosis groups^22^. Finally, regarding the data analyzed in this study, it was largely medical doctors who prescribed the drugs or medical procedures documented in the database depending on what they regarded as comorbid conditions; therefore, in some percent of cases, the treatments undertaken might not directly reflect the disease status. In this regard, Ishii *et al*.^42^ discussed the possibility of underestimating disease prevalence in the context of a clinical setting, for example, regarding osteoporosis or Alzheimer’s disease. However, our results clearly address the more common acute comorbidities among hospitalized elderly patients in Japan. Aoki *et al*.^43^ reported that five comorbid patterns were observed in a nationwide cross-sectional survey of 3,256 adult Japanese residents: cardiovascular/renal/metabolic, neuropsychiatric, skeletal/articular/digestive, respiratory/dermal, and malignant/digestive/urologic. Our study targeted the elderly population; however, the comorbid patterns were, in part, similar to those in the study results of Aoki *et al*.^43^ For example, we did not find the skeletal/digestive or respiratory/dermal comorbid pattern; one possible explanation for the difference is disease selection and categorization. Moreover, we did not include dermal diseases and focused on specific diseases, such as pneumonia and dementia, not on a broad category of neuropsychiatric or respiratory diseases.

Despite these limitations, our study has several strong points. The NDB database analyzed in this study is nationally representative and contains data on approximately 4 million patients. Most Japanese citizens must belong to one of the major insurance systems, including the National Health Insurance, the Japan Health Insurance Association, health insurance arrangements provided by unions, employee insurance provided by mutual aid associations, and the Medical Care System for the Elderly, which covers people aged ≥75 years^12^. The NDB database includes all the processed health-care insurance claims, so it is highly representative of the country as a whole.

From a methodological perspective, our study used factor analysis to identify comorbidity patterns among elderly patients in Japan. This approach worked well for our dataset. The approach generated a clear but limited set of five factors for both sexes and produced a good model fit, as reflected by a high rate of cumulative percentage of variance and a more than sufficient sampling adequacy, based on the KMO measure.

A better understanding of the prevalence of different comorbid conditions among the elderly population may help devise appropriate health policy strategies. In particular, the prevalence of multimorbid conditions that require pharmacotherapy drives the use of multiple medications among older adults. As Arai *et al*.^29^ proposed the need for “multidisciplinary care” to meet the various demands for medical care and welfare of elderly individuals, our suggestion for the practical implication of this study is that health-care professionals should use a holistic approach by being aware of the physical traits of older people who suffer from multimorbid status, including dementia and geriatric syndromes, such as depression, falls, and urinary incontinence. Our findings revealed that cardiovascular diseases are the leading factors that result in a comorbid status; furthermore, some sex differences in morbidity status were observed regarding DPC hospitalizations throughout Japan, with musculoskeletal diseases being especially prominent in elderly female patients. Recognizing comorbidities might require methods that complement disease-specific and reductionist approaches. However, our results do not address the realities of comorbidities that may exist in the general elderly population, including the outpatient population; therefore, further research focusing on older adult outpatients and using longitudinal analysis is warranted.

## Methods

The NDB database contains comprehensive claim records regarding DPC inpatient care within the National Health Insurance system of Japan, and the data available include the insurer’s code and the insured’s ID number, diagnosis, age, sex, date of outpatient service, date of admission, date of discharge, procedures undertaken, drug information, etc.^10^. In this study, we used datasets based on inpatient claims. All claim data used in our analysis were deidentified by the Ministry of Health, Labour and Welfare, and the guidelines on information security from the ministry were followed in the study. To use the NDB database, the opt-out method was applied in the Health and Welfare Ministry in Japan, and inspection by and permission from the ministry for publication are needed before the submission of the draft to ensure that patient privacy is respected. All claim records regarding the admission of patients aged >60 years with a diagnosis of the various lifestyle-related diseases shown in Table 1 were identified from the NDB database. In the 2015 fiscal year (from April 2015 to March 2016), we enrolled any patients who had at least one diagnosis with any treatment processed in the claim data. Under the DPC approach for identifying illnesses and their various components, providers are reimbursed for basic hospital stays, tests, diagnostic imaging, medication, and injections, as well as for treatments costing less than 1,000 “points” (currently 10,000 yen, or about $125). Our data included only DPC claim data and therefore excluded any claims generated in various fee-for-service systems^13^. All included diagnoses were categorized according to the “The International Classification of Disease, 10th Revision, Clinical Modification” (ICD-10) diagnosis system.

Comorbid health conditions such as heart and pulmonary diseases, diabetes, and arthritis are commonly present in elderly patients. Either at least one claim with a diagnosis during the 2015 fiscal year or one claim with a diagnosis for a procedure was required to classify patients as having the targeted diseases. In our study, prevalence values were calculated for the total DPC cohort among hospitalized patients during the 2015 fiscal year. It is important to accurately apply each diagnostic code, and this depends upon the type of disease as well as the reality that in Japan, doctors often use the insurance disease name as the suspected disease name. Therefore, we used only the main diagnostic codes, the subdiagnostic codes, and the medical resource codes among all the DPC inpatient disease codes, which are inspected by the Ministry of Health, Labour and Welfare. The institutional review board of the Juntendo University Hospital approved this study (registration number: 15–178).

### Data analysis

Our study focused on the trends in the prevalence of medical conditions, following the definition of lifestyle-related diseases provided by the Ministry of Health, Labour and Welfare, which includes diabetes, hypertension, hyperlipidemia, liver dysfunction, cerebrovascular illness, cardiovascular diseases, artery dissection, and renal dysfunction. The patient survey in 2017 reported that the most common cause of disability was cerebrovascular accidents, followed by dementia, frailty due to aging, joint disorders, bone fracture, and cardiac disease^14^. In addition, the standard core recommendations used in normal systematic multimorbidity reviews guided our selection and definition of morbidities included in this study. For the principal factor analysis, the selection and definition of morbidities were partly subjective, and we selected 25 diseases in this regard: myocardial infarction (or acute myocardial infarction), CHF (or acute CHF), cardiac arrhythmia/atrial fibrillation, artery dissection/aortic aneurysm, depression, schizophrenia, cerebrovascular diseases, dementia, COPD, pneumonia, cancer, hypertension, dyslipidemia, diabetes mellitus, thyroid disease, rheumatoid arthritis, knee arthritis, allergy, renal failure, hip fracture, osteoporosis, digestive diseases, cataract^12^, lymphoma and leukemia, and Parkinson’s diseases. In the analysis of disease transition by age, we selected the main causes of death in Japan, such as cardiovascular diseases, cerebrovascular diseases, pneumonia, cancer, and renal failure.

We stratified the data by sex and examined the trends in the prevalence rates of the selected medical conditions across the seven 5-year age strata including individuals over the age of 60 years (60–64, 65–69, 70–74, 75–79 80–84, 85–89, 90–94, 95–99 and ≥100). For the denominator in the prevalence calculations, we used the total number of DPC hospital patients, not the total population in Japan, and thus, the prevalence reported in this study is not the population prevalence. Correlations among diagnosis groups were analyzed using exploratory factor analysis. The principal factor analysis method was used to identify sets of disease groups with a common underlying causal factor. The extraction of the initial solution was performed using the principal factor method with squared multiple correlations for the prior communality estimates. Our final dataset of morbidities was coded in binary format (0 = no disease, 1 = presence of disease), and the correlation matrix among the diagnoses was computed using tetrachoric correlation^36^. The adequacy of the sample was analyzed by measuring the KMO statistic, which gets closer to 1 with a greater goodness of fit; in addition, the proportion of cumulative variance was described for variability. The promax rotation was applied for accurate estimation in primary factor analysis. We included patients without the aforementioned diseases in our study to prevent the overestimation of correlations among diagnosis groups. The number of factors to be extracted was determined using scree plots and the clinical evaluation of the different solutions obtained^44,45^. Diseases with a factor loading higher than 0.20 were selected, with the aim of determining the diseases that composed each pattern. Although comorbidities are usually defined as the simultaneous presence of two or more chronic diseases, our definition more specifically required the presence of more than one chronic disease in 1 study year, as recorded in the DPC claim data. An SQL server was used for data extraction, and SAS 9.4 software was used to perform statistical analysis.

## Data Availability

Data that support the findings of this study are available from the Ministry of Health, Labour and Welfare. However, restrictions apply to the availability of these data, as they are used under license only for current study projects; therefore, they are not publicly available.
